# Metagenomic next-generation sequencing for *Mycobacterium tuberculosis* complex detection: a meta-analysis

**DOI:** 10.3389/fpubh.2023.1224993

**Published:** 2023-08-11

**Authors:** Yulian Li, Wentao Bian, Shiping Wu, Jie Zhang, Dan Li

**Affiliations:** ^1^College of Medical Technology, Chengdu University of Traditional Chinese Medicine, Chengdu, China; ^2^Affiliated Hospital of North Sichuan Medical College, Nanchong, China; ^3^Department of Laboratory Medicine, Sichuan Provincial People's Hospital, University of Electronic Science and Technology of China, Chengdu, China; ^4^School of Medicine, University of Electronic Science and Technology of China, Chengdu, China

**Keywords:** metagenomic next-generation sequencing, tuberculosis, clinical specimen, infectious disease, mixed infections

## Abstract

**Objective:**

Metagenomic next-generation sequencing (mNGS) has been gradually applied to the diagnosis of tuberculosis (TB) due to its rapid and highly sensitive characteristics. Despite numerous studies on this subject, their results vary significantly. Thus, the current meta-analysis was performed to assess the performance of the mNGS on tuberculosis.

**Methods:**

PubMed, Embase, Web of Science, and The Cochrane Library were searched up to June 21, 2023. Studies utilizing the mNGS for tuberculosis detection were included. The risk of bias was assessed by QUADAS-2, and a meta-analysis was performed with STATA14.0 software.

**Results:**

Seventeen studies comprising 3,205 specimens were included. The combined sensitivity and specificity of mNGS for clinical specimens were 0.69[0.58–0.79] and 1.00[0.99–1.00], respectively. Subgroup analysis identified sequencing platform, diagnostic criteria, study type, sample size, and sample types as potential sources of heterogeneity. Cerebrospinal Fluid (CSF) has a lower sensitivity of 0.58 (0.39–0.75). In a population with a 10% prevalence rate, the accuracy of sensitivity reached 94%.

**Conclusion:**

Metagenomic next-generation sequencing technology exhibits high sensitivity and speed in diagnosing *Mycobacterium tuberculosis*. Its application in mono and mixed infections peoples shows promise, and mNGS is likely to be increasingly used to address challenges posed by *Mycobacterium tuberculosis* complexes in the future.

## Introduction

Prior to the new coronavirus pandemic, TB represented the most significant burden of human infectious disease, with roughly 10 million new cases and 1.5 million deaths per year (1). The emergence of multi-drug-resistant tuberculosis (MDR-TB) and extensively drug-resistant tuberculosis (XDR-TB) due to the long-term use of isoniazid, rifampicin, and quinolones, has increased treatment difficulties and patient harm (2). Existing diagnostic methods for tuberculosis struggle to simultaneously offer rapid diagnosis and high precision. While the GeneXpert MTB/RIF test is a rapid and high-precision diagnostic method, it primarily detects *Mycobacterium tuberculosis* complexes and lacks the ability to diagnose multiple infections. In recent years, the metagenomic next-generation sequencing (mNGS) has been introduced as a new diagnostic method for detecting pathogens. mNGS provides unbiased pathogen detection directly from clinical specimens, and generates up to billions of reads in a single run, allowing comprehensive analysis of all base sequences in clinical specimens (3).

mNGS’s rapid diagnosis and high precision capabilities enable it not only to detect *Mycobacterium tuberculosis*, but also other potential pathogens, increasing the diagnostic accuracy in mixed infections (4). Compared to the GeneXpert MTB/RIF test, mNGS offers a clearer diagnosis in patients with severe symptoms [A proportion of severely infected patients present with *Mycobacterium tuberculosis* complexes combined with other microorganisms (5)]. Many cases have been reported where infectious agents were directly detected from body fluid specimens, such as cerebrospinal fluid and blood using next-generation sequencing technology (6–8). Although recent studies have reported the potential application of metagenomic Next-Generation Sequencing in diagnosing *Mycobacterium tuberculosis* complexes, the reported sensitivity and specificity vary across studies, necessitating a systematic review and meta-analysis.

This study performed a meta-analysis of the literature regarding metagenomic next-generation sequencing technology for the detection of *Mycobacterium tuberculosis* complexes in clinical specimens. We aimed to validate the diagnostic performance of metagenomic next-generation sequencing in detecting *Mycobacterium tuberculosis* complexes in clinical specimens. This study evaluated existing studies to derive consolidated conclusions regarding sensitivity and specificity to explore its true diagnostic value.

## Method

### Registration and ethics

The study was conducted in accordance with the PRISMA 2020 statement (9) and the protocol was registered on the International Platform for Systematic Reviews (ID: CRD42023400281).

### Search strategy

We conducted a comprehensive search in the four major databases: PubMed, Embase, Web of Science, and The Cochrane Library, using the terms “tuberculosis” and “high-throughput nucleotides” and combine them with free words for a comprehensive search. The search was independently performed by two researchers, Yulian Li and Wentao Bian, with the consolidated results being aggregated. The full search strategy can be found in Supplementary Material S1.

### Study selection

Inclusion criteria encompassed randomized trials, cohort or case–control studies, that utilized the metagenomic next-generation sequencing platform (mNGS) on clinical specimens with non-tuberculosis specimens serving as controls. We excluded animal experiments, reviews, conference summaries, case reports, non-English literatures and non-SCI academic journals. There were no restrictions on the year of publication.

Eligible populations included: (1) individuals suspected to be infected with the *Mycobacterium tuberculosis* complexes by clinicians; (2) Individuals who collect specimens prior to antituberculosis treatment as much as possible; (3) patients with data available through electronic medical records (routine data, such as sex, age, bacteriological or imaging examination, and whether they received anti-tuberculosis drug treatment). The index test was the detection of DNA in clinical specimens from suspected tuberculosis patients via the mNGS platform. Reference criteria included clinical symptoms, imaging reports, bacteriological reports, and anti-TB drug efficacy. According to guidelines jointly developed by the United States Centers for Disease Control and Prevention and the American Society for Infectious Diseases (10), specimens from patients with positive results other than mNGS were considered TB patient specimens. Specimens from patients with negative results on all tests except mNGS were considered non-tuberculosis patient specimens.

Two researchers (YL, WT) independently screened search results based on titles and abstracts. Any disagreements were resolved by a third researcher (JZ).

### Data extraction

A 2 × 2 table was used to extract data from the studies: true positive, false positive, true negative, and false negative. Additional extracted data included country, research type, sample size, sequencing platform, sample type, and average age of patients. The process was carried out independently by two researchers (YL, WT), with any disagreements resolved by a third researcher (JZ).

### Risk of bias assessment

The risk of bias in diagnostic accuracy was assessed using the QUADAS-2 tool (11). One researcher (YL) independently evaluated all included studies. This assessment was reviewed by another reviewer (WT), and any disagreements were resolved by a third researcher (JZ).

### Data analysis

The primary outcome was a summary of the combined sensitivity and specificity of all studies. Secondary outcomes included subgroup analysis based on sequencing platform, diagnostic criteria, study type, sample size, and sample types based on pre-collected information. The grouping information was defined as different subgroups and added to the 2 × 2 table. Choose random or fixed effects models depending on the magnitude of heterogeneity. To evaluate secondary outcomes, at least four studies were required to calculate the sensitivity and specificity of previously defined subgroup variables. The *Q*-test *p-*value in the forest map was used to test for heterogeneity, with *p* < 0.05 considered statistically significant. *I^2^* was used to describe the size of heterogeneity, and *I^2^* > 50% was considered significant heterogeneity.

The subjects of the diagnostic studies were each sample collected from patients suspected of infection (including multiple specimens from the same individual). A receiver operating characteristic (ROC) curve was plotted to assess the average accuracy across all possible thresholds. A diagnostic value was considered high if the area under the curve (AUC) was greater than 0.9. To assess the stability of the results, a sensitivity analysis was performed using a combination of sensitivity and specificity after removing studies at high risk of bias. In addition, a Feigen diagram, plotted according to the disease prevalence rate, was used to evaluate the real-world effect of the diagnostic study. Due to the exclusion of certain studies, discrepancies may exist between the published studies and those not included in the analysis. To account for this, a publication bias test was performed using the funnel chart. *p-*values greater than 0.05 were considered not to have statistically significant publication bias. STATA14 software was used to analyze the data.

## Results

### Summary of study results

**Figure 1:**
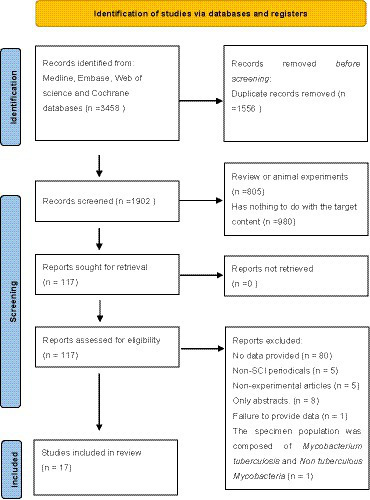
Research and selection process.

A total of 3,458 articles (comprising 1902 unique records) were retrieved from the database. Following a thorough review of these articles, 17 were included in the meta-analysis (12–28) (Figure 1). The basic characteristics of all included studies are shown in Table 1. All studies were conducted in China, with 13 retrospective studies and 4 prospective studies. The age range of tuberculosis patients in these studies was between 30 and 60 years old. Most studies were sequenced using the BGISEQ platform, with the read length ranging from 200 to 300 bp. Several studies used multiple specimens from the same patient to evaluate sensitivity and specificity. The specimens are divided into pulmonary specimens and extrapulmonary specimens. The study of pulmonary specimens focuses on bronchoalveolar lavage fluid, and extrapulmonary specimens mainly focus on cerebrospinal fluid. Negative specimens for evaluation included those infected with other pathogens or non-infectious specimens, which were identified through diagnostic methods other than mNGS. The final reference criteria was made by clinicians according to clinical guidelines, which required at least one positive indicator except for mNGS.

**Table 1:** The characteristics of included studies.

Study (First author/year)	DNA sequencing platform	Country	Sample size	Specimen type	Type of research	Confirmed MTBC positive	Confirmed MTBC negative
Chen 2020 (12)	BGISEQ-50	C	200-300 bp	Pulmonary and extrapulmonary specimen	Retrospective	24 (66.7%)	33 (97.1%)
Fu 2022 (13)	MGISEQ-2000	C	200-300 bp	BALF and biopsy tissue	Retrospective	36 (78.3%)	357 (100%)
Jin 2020 (14)	BGISEQ-100	C	150-200 bp	Pulmonary and extrapulmonary specimen	Retrospective	62 (49.6%)	683 (98.3%)
Liu 2021 (15)	BGISEQ-50	C	NA	BALF	Retrospective	85 (59.9%)	111 (100%)
Shi 2020 (16)	NextSeq CN500	C	200-300 bp	BALF	Prospective	23 (47.9%)	61 (98.3%)
Xu P. 2022 (17)	NextSeq 550 DX	C	NA	BALF	Retrospective	67 (94.4%)	23 (100%)
Zhou 2019 (18)	BGISEQ-50	C	NA	Pulmonary and extrapulmonary specimen	Prospective	20 (44%)	59 (98%)
Zhu 2021 (19)	BGISEQ-50	C	NA	BALF and biopsy tissue	Retrospective	41 (89.1%)	60 (98.4%)
Sun 2021 (20)	BGISEQ-50	C	200–300 bp	Extrapulmonary specimen	Retrospective	101 (56.1%)	28 (100%)
Li 2022 (21)	MGISEQ-2000	C	200-300 bp	granulation tissue and pus	Prospective	36 (94.7%)	62 (100%)
Jin 2023 (22)	BGISEQ-50	C	NA	spinal tissue	Retrospective	79 (71.2%)	92 (100%)
Gao 2023 (23)	NA	C	NA	BALF	Retrospective	30 (79.0%)	148 (100%)
Chen 2022 (24)	BGISEQ-100 BGISEQ-50/MGISEQ-2000	C	200–300 bp	CSF	Retrospective	74 (63.2%)	99 (100%)
Yu 2021 (25)	NA	C	NA	CSF	Retrospective	10 (43.5%)	14 (100%)
Yan 2020 (26)	BGISEQ-50	C	NA	CSF	Retrospective	38 (84.4%)	6 (100%)
Wang 2019 (27)	BGISEQ-100	C	200 ~ 300 bp	CSF	Retrospective	18 (78.3%)	6 (100%)
Xing 2020 (28)	BGISEQ-500/50	C	NA	CSF	Prospective	12 (27.3%)	163 (96.4%)

C, China; NA, unable to get data; BALF, bronchoalveolar lavage fluid obtained by bronchoscope; Pulmonary specimen: includes BALF, Sputum and biopsy tissue etc;

Extrapulmonary specimen includes Serous fluid, Pus, extrapulmonary tissue, Blood and Cerebrospinal fluid etc.

### Risk of bias assessment

**Figure 2:**
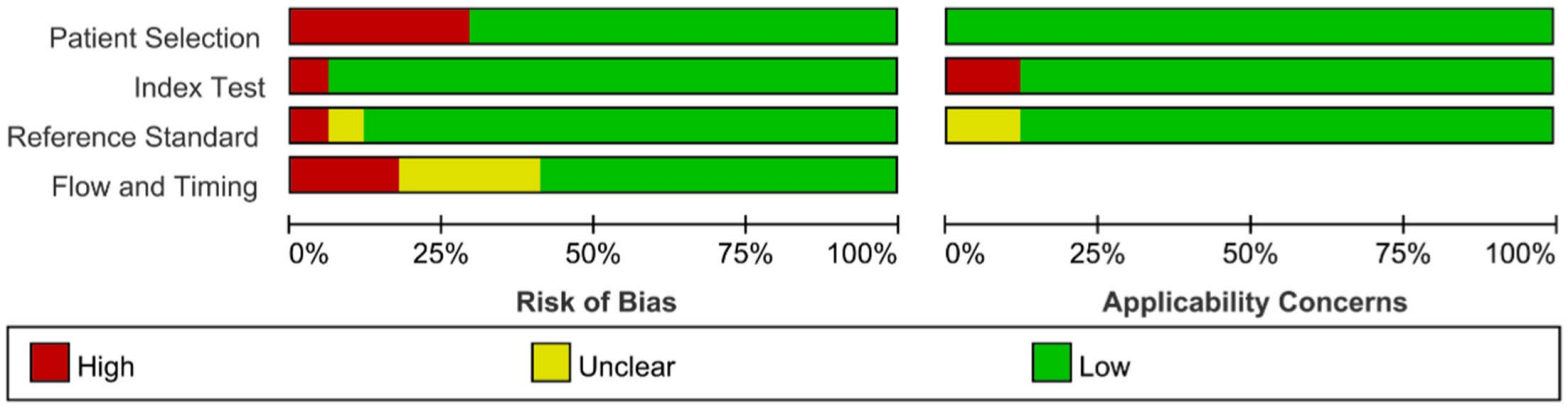
Risk of bias assessment evaluation chart.

Figure 2 presents the results of the risk of bias assessment. Eleven studies exhibited a low risk of bias, whereas 6 demonstrated a high risk of bias.

### Data analysis

**Figure 3:**
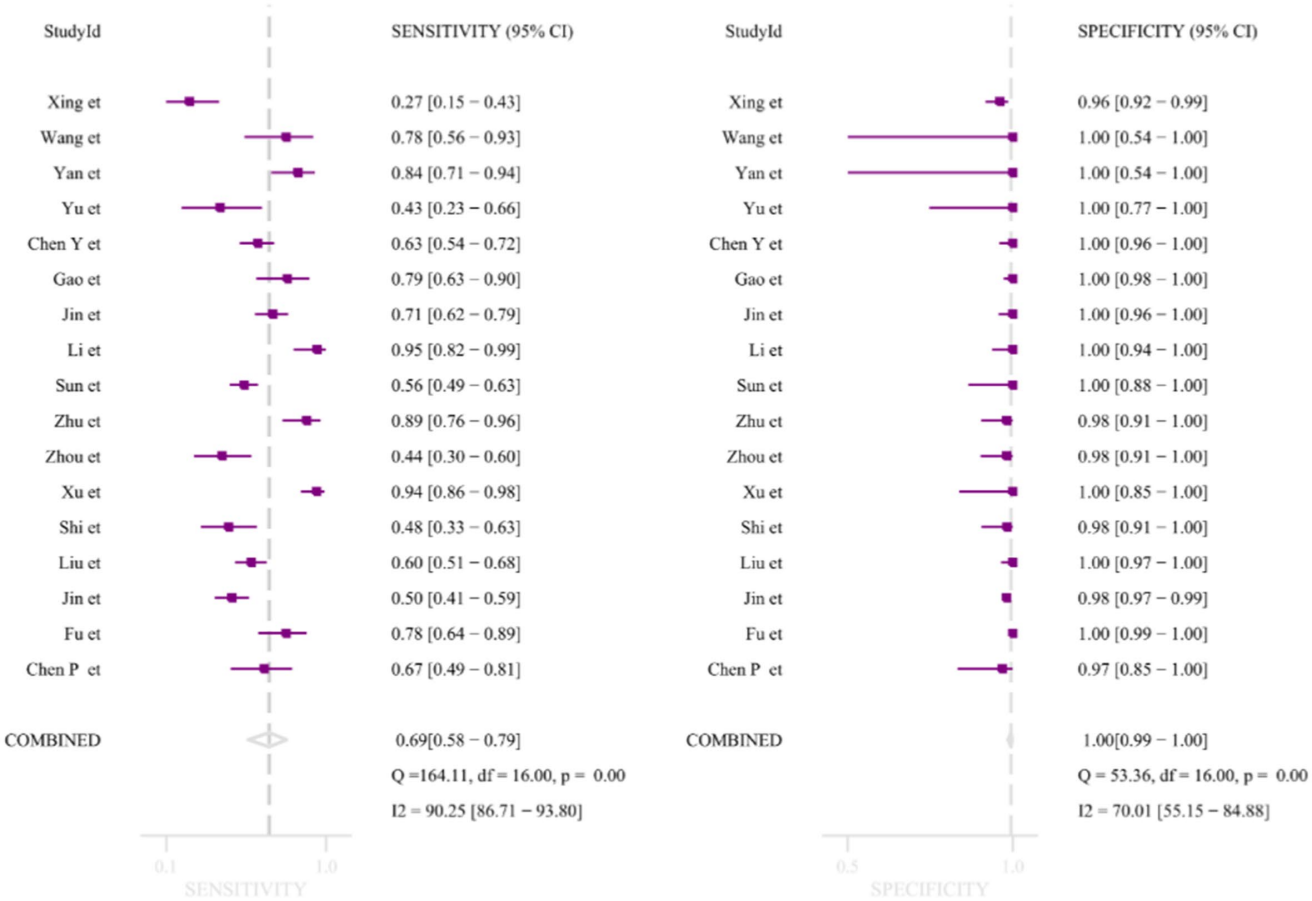
Meta-analysis of sensitivity and specificity.

A total of 3,205 specimens were collected from the 17 studies. The combined sensitivity and specificity of the summarized primary results were 0.69 [0.58–0.79] and specificity 1.00 [0.99–1.00] respectively (Figure 3). Because there were 5 studies with a high risk of bias in the patient domain, 3 studies with a high risk of bias in the test domain, 3 studies with an unknown risk of bias in the reference domain, and 7 studies with unclear case flow and timing domains or High risk and large bias, moderately conclusive evidence downgraded (12, 13, 15, 19, 22–26). Supplementary Table S2 shows the subgroup analysis of potential sources for evaluating sensitivity and specificity. The results indicated that mNGS had high sensitivity and specificity but significant heterogeneity (*p* < 0.05). In the subgroup analysis of specimen types, the sensitivity of pulmonary specimens (0.75) > extrapulmonary specimens (0.61). Interestingly, when the specimen was BALF (bronchoalveolar lavage fluid), the sensitivity was (0.75), which may be due to the fact that BALF was the main clinical specimen in the study of pulmonary specimens. The sensitivity was lowest (0.58) when the specimen was CSF (cerebrospinal fluid). The sensitivity was highest (0.78) when studies with sample sizes <100 were included. However, the sensitivity was lowest when all prospective studies were included for analysis (0.58). Regarding specificity, in the subgroup analysis of specimen types, the specificity was (0.99–1.00) with a slight heterogeneity [48.84–66.52]. When specimens were BALF, specificity heterogeneity was not significant (*I^2^* = 48.84, *p* = 0.08). Heterogeneity in specificity was not significant when all studies with less than 100 specimens were included, although the results require caution (*I^2^* = 0.00, *p* = 0.66). To conclude, the heterogeneity of specificity (0.99) was not significant when all studies using the BGISEQ-50 platform were included (*I^2^* = 8.49, *p* = 0.36).

**Figure 4:**
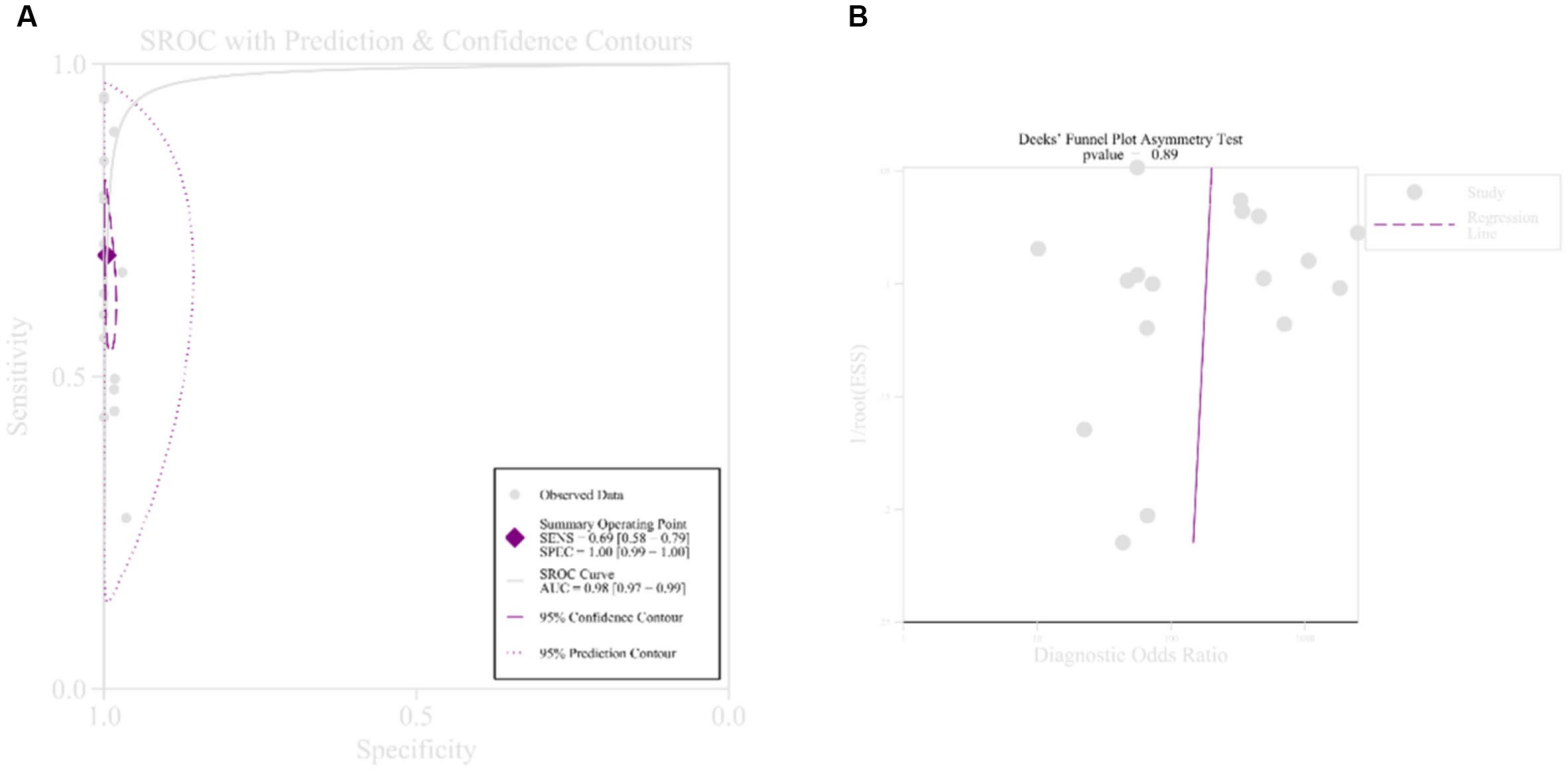
Receiver operating curve and publication bias. **(A)** Receiver operating curve; **(B)** publication bias.

All subgroup sensitivities showed significant heterogeneity according to previously defined subgroup variables: sequencing platform, diagnostic criteria, study type, sample size, and specimen type, and in terms of specificity, all subgroup specificities showed significant heterogeneity except for the BALF, BGISEQ-50, and sample < 100 subgroups, which may be the source of heterogeneity. In terms of measuring average accuracy at all possible thresholds, the receiver operating characteristic (Figure 4A) area under the curve was 0.98 (>0.9). The publication bias plot was symmetrical as a whole (Figure 4B), and the publication bias was not significant (*p* = 0.89). Finally, even with the exclusion of six studies with a high risk of bias, heterogeneity in sensitivity and specificity persisted.

According to the World Health Organization’s reported TB incidence of 0.055% in 2022 (29), the accuracy of Fagan’s chart sensitivity was 8%, and the specificity error rate was 0% (Supplementary Figure S2A). When using an environment with a prevalence of 10%, the accuracy of sensitivity was 94% and the specificity error rate was as low as 3% (Supplementary Figure S2B).

## Discussion

Due to variations in the outcomes of past studies, the present meta-analysis, which collated results from 17 literatures, based on current data found that mNGS had the potential to diagnose TB in clinical specimens with high sensitivity and specificity 0.69 [0.58–0.79] and 1.00 [0.99–1.00]. Pulmonary specimens might be more diagnostic than extrapulmonary specimens. In areas of high TB incidence, its efficacy in diagnosing TB is noteworthy. Finally, the promise of mNGS in diagnosing patients with mixed infections was observed.

Diagnosing *Mycobacterium tuberculosis* complexes is notoriously challenging, often resulting in delayed treatment for patients. Rapid and sensitive diagnostic methods are needed. mNGS is a very effective diagnostic tool, outperforming traditional methods such as culture and acid-fast staining (1). Tuberculosis is mainly airborne (30), and in our study, we found that pulmonary specimens were better diagnostic than extrapulmonary specimens. Even the BALF samples were diagnosed more effectively than conventional diagnostic methods. In CSF specimens, the diagnosis was slightly less effective, but of some value in diagnosing tuberculous meningitis, with results similar to those of a previous meta-analysis of CSF (31). According to Dowdy et al., current experimental methods are not sufficient to diagnose all patients with TB. In high-burden countries, TB prevalence may be as high as 10% (32). The inclusion of TB prevalence in the analysis yielded sensitivity accuracy of 94% and specificity error rates as low as 3%, Supporting the use of mNGS by clinicians to screen patients suspected of *Mycobacterium tuberculosis* complexes infection. Chen et al. (12) eventually detected samples of *Mycobacterium tuberculosis* complexes and other microorganisms, and previous cases have also reported the diagnostic value of mNGS in patients with mixed infections (33). Thresholds for all studies were determined in advance, random-effects models were used to summarize all results and SROAUC was used to evaluate the diagnostic effects of mNGS experiments. Subgroup analysis and sensitivity analysis were used to explore the robustness of the results. Some studies had incomplete information, and thus the mean age and gender of patients were not included in the analysis.

Although the sequencing platform, diagnostic criteria, study type, sample size and sample type were all considered as potential sources of heterogeneity in our study, heterogeneity could not be fully explained. This may be related to library construction capabilities and pre-treatment of specimens (The thick cell wall of *Mycobacterium tuberculosis* complexes requires the pre-addition of reagents to disrupt the cell wall release of nucleic acid). The efficiency of nucleic acid extraction can vary among different methods (34). In addition, the time of specimen obtaining was not described in detail in most of the studies. The findings should be interpreted with caution due to the heterogeneity of the sensitivity and specificity pooled results, the limited number of studies and the fact that most studies focused on BALF and CSF specimens. This study is mainly a retrospective study, with a lower diagnostic efficacy demonstrated in prospective studies, emphasizing more prospective studies.

This research has some limitations. Six studies had a high risk of bias, five studies’ patients were discontinuous, posing a high risk of bias, and clinical characteristics of patients (gender, age, anti-tuberculosis drug use) were not analyzed due to incomplete information. As the study focused on BALF and CSF, although no subgroup analysis was performed, a low sensitivity was observed for sputum in pulmonary specimens (30.3%) and a high sensitivity for granulation tissue and pus in extra-pulmonary specimens (94.7%), although individual studies were not representative. This emphasizes the need for more studies with different types of clinical specimens. Furthermore, when incorporating the actual prevalence rate in China (29), the accuracy of the results decreased. Metagenomic Next-Generation sequencing is an expensive diagnostic tool, which may increase the financial burden on patients, and some patients may not have access to specimens with high bacterial content due to their condition limiting the adoption of this method. The diagnosis of tuberculosis requires a comprehensive reference standard for the clinician, and mNGS should be used only when the patient has a high suspicion of tuberculosis infection and specimens are available, as long as economic conditions allow.

In conclusion, this study pooled all previous results to assess the potential of mNGS for the diagnosis of TB in clinical specimens. In addition, it found that mNGS can be used in areas of high prevalence and patients with mixed infections, contributing to the comprehensive diagnosis of TB. In light of the 10-year setback in TB control due to COVID-19, the need for new diagnostics like mNGS is critical.

## Conclusion

Metagenomic next-generation sequencing technology can increase the efficiency of diagnosing tuberculosis (TB) in clinical specimens and has shown significant efficacy, especially in areas with high TB prevalence. This approach is recommended as an additional to diagnose TB when patient specimens are available and when economic conditions allow. Future studies should extend the evaluation to different countries and regions and evaluate various types of clinical specimens, with a focus on severely infected and older adult patients.

## Data availability statement

The original contributions presented in the study are included in the article/Supplementary material, further inquiries can be directed to the corresponding author.

## Author contributions

JZ designed the study. YL and WB contributed to the manuscript writing and revision. All authors contributed to the article and approved the submitted version.

## Conflict of interest

The authors declare that the research was conducted in the absence of any commercial or financial relationships that could be construed as a potential conflict of interest.

## Publisher’s note

All claims expressed in this article are solely those of the authors and do not necessarily represent those of their affiliated organizations, or those of the publisher, the editors and the reviewers. Any product that may be evaluated in this article, or claim that may be made by its manufacturer, is not guaranteed or endorsed by the publisher.
