# Supplementary material for: Metagenomic next-generation sequencing for Mycobacterium tuberculosis complex detection: a meta-analysis
Source: Front Public Health. 2023 Aug 11;11:1224993. doi: 10.3389/fpubh.2023.1224993 (PMC10450767; doi:10.3389/fpubh.2023.1224993)
Supplement: Supplementary file 1 [file Data_Sheet_1.DOCX]

**Pubmed：**

**1#** (((((((((Tuberculosis) OR (Tuberculoses)) OR (Kochs Disease)) OR (Koch's Disease)) OR (Koch Disease)) OR (Mycobacterium tuberculosis Infection)) OR (Infection, Mycobacterium tuberculosis)) OR (Infections, Mycobacterium tuberculosis)) OR (Mycobacterium tuberculosis Infections)) AND (((((((((((((((((((((((((((((High Throughput Nucleotide Sequencing) OR (Nucleotide Sequencing, High-Throughput)) OR (Sequencing, High-Throughput Nucleotide)) OR (Next-Generation Sequencing)) OR (Next Generation Sequencing)) OR (Sequencing, Next-Generation)) OR (Illumina Sequencing)) OR (Sequencing, Illumina)) OR (Ion Torrent Sequencing)) OR (Sequencing, Ion Torrent)) OR (Ion Proton Sequencing)) OR (Sequencing, Ion Proton)) OR (Deep Sequencing)) OR (Sequencing, Deep)) OR (High-Throughput RNA Sequencing)) OR (High Throughput RNA Sequencing)) OR (RNA Sequencing, High-Throughput)) OR (Sequencing, High-Throughput RNA)) OR (Massively-Parallel Sequencing)) OR (Massively Parallel Sequencing)) OR (Sequencing, Massively-Parallel)) OR (Pyrosequencing)) OR (High-Throughput Sequencing)) OR (High Throughput Sequencing)) OR (Sequencing, High-Throughput)) OR (High-Throughput DNA Sequencing)) OR (DNA Sequencing, High-Throughput)) OR (High Throughput DNA Sequencing)) OR (Sequencing, High-Throughput DNA))

Total results:1231

**Web of science：**

**1#** TS=(( Tuberculosis OR Tuberculoses OR Kochs Disease OR Koch's Disease OR Koch Disease OR Mycobacterium tuberculosis Infection OR Infection, Mycobacterium tuberculosis OR Infections, Mycobacterium tuberculosis OR Mycobacterium tuberculosis Infections))AND TS=(( High Throughput Nucleotide Sequencing OR Nucleotide Sequencing, High-Throughput OR Sequencing, High-Throughput Nucleotide OR Next-Generation Sequencing OR Next Generation Sequencing OR Sequencing, Next-Generation OR Illumina Sequencing OR Sequencing, Illumina OR Ion Torrent Sequencing OR Sequencing, Ion Torrent OR Ion Proton Sequencing OR Sequencing, Ion Proton OR Deep Sequencing OR Sequencing, Deep OR High-Throughput RNA Sequencing OR High Throughput RNA Sequencing OR RNA Sequencing, High-Throughput OR Sequencing, High-Throughput RNA OR Massively-Parallel Sequencing OR Massively Parallel Sequencing OR Sequencing, Massively-Parallel OR Pyrosequencing OR High-Throughput Sequencing OR High Throughput Sequencing OR Sequencing, High-Throughput OR High-Throughput DNA Sequencing OR DNA Sequencing, High-Throughput OR High Throughput DNA Sequencing OR Sequencing, High-Throughput DNA))

Total results:1042

**Cochrane**

**#1** High Throughput Nucleotide Sequencing

**#2** Illumina Sequencing OR Ion Torrent Sequencing OR Ion Proton Sequencing OR Deep Sequencing OR High-Throughput RNA Sequencing OR High Throughput RNA Sequencing OR Massively-Parallel Sequencing OR Massively Parallel Sequencing OR Pyrosequencing OR High-Throughput Sequencing OR High Throughput Sequencing OR High-Throughput DNA Sequencing OR High Throughput DNA Sequencing

**#3** Tuberculosis

**#4** Tuberculosis OR Kochs Disease OR Koch's Disease OR Koch Disease OR Mycobacterium tuberculosis Infection OR Infection, Mycobacterium tuberculosis OR Infections, Mycobacterium tuberculosis OR Mycobacterium tuberculosis Infections#5 **#1** OR **#2**

**#6** #3 OR #4

**#7** #5 AND #6

Total results:25

**Embase**

**#1** High Throughput Nucleotide Sequencing

**#2**‘High Throughput Nucleotide Sequencing’:ab,ti OR ‘Nucleotide Sequencing, High-Throughput’:ab,ti OR ‘Sequencing, High-Throughput Nucleotide’:ab,ti OR ‘Next-Generation Sequencing’:ab,ti OR ‘Next Generation Sequencing’:ab,ti OR ‘Sequencing, Next-Generation’:ab,ti OR ‘Illumina Sequencing’:ab,ti OR ‘Sequencing, Illumina’:ab,ti OR ‘Ion Torrent Sequencing’:ab,ti OR ‘Sequencing, Ion Torrent’:ab,ti OR ‘Ion Proton Sequencing’:ab,ti OR ‘Sequencing, Ion Proton’:ab,ti OR ‘Deep Sequencing’:ab,ti OR ‘Sequencing, Deep’:ab,ti OR ‘High-Throughput RNA Sequencing’:ab,ti OR ‘High Throughput RNA Sequencing’:ab,ti OR ‘RNA Sequencing, High-Throughput’:ab,ti OR ‘Sequencing, High-Throughput RNA’:ab,ti OR ‘Massively-Parallel Sequencing’:ab,ti OR ‘Massively Parallel Sequencing’:ab,ti OR ‘Sequencing, Massively-Parallel’:ab,ti OR ‘Pyrosequencing’:ab,ti OR ‘High-Throughput Sequencing’:ab,ti OR ‘High Throughput Sequencing’:ab,ti OR ‘Sequencing, High-Throughput’:ab,ti OR ‘High-Throughput DNA Sequencing’:ab,ti OR ‘DNA Sequencing, High-Throughput’:ab,ti OR ‘High Throughput DNA Sequencing’:ab,ti OR ‘Sequencing, High-Throughput DNA’:ab,ti

**#3** Tuberculosis

**#4** ‘Tuberculoses’:ab,ti OR ‘Kochs Disease’:ab,ti OR ‘Koch Disease’:ab,ti OR ‘Mycobacterium tuberculosis Infection’:ab,ti OR ‘Infection, Mycobacterium tuberculosis’:ab,ti OR ‘Infections, Mycobacterium tuberculosis’:ab,ti OR ‘Mycobacterium tuberculosis Infections’:ab,ti

**#5** #1 OR #2

**#6** #3 OR #4

**#7** #5 AND #6

Total results:1160
