# Supplementary material for: Metagenomic next-generation sequencing for Mycobacterium tuberculosis complex detection: a meta-analysis
Source: Front Public Health. 2023 Aug 11;11:1224993. doi: 10.3389/fpubh.2023.1224993 (PMC10450767; doi:10.3389/fpubh.2023.1224993)
Supplement: Supplementary file 2 [file Data_Sheet_2.DOCX]

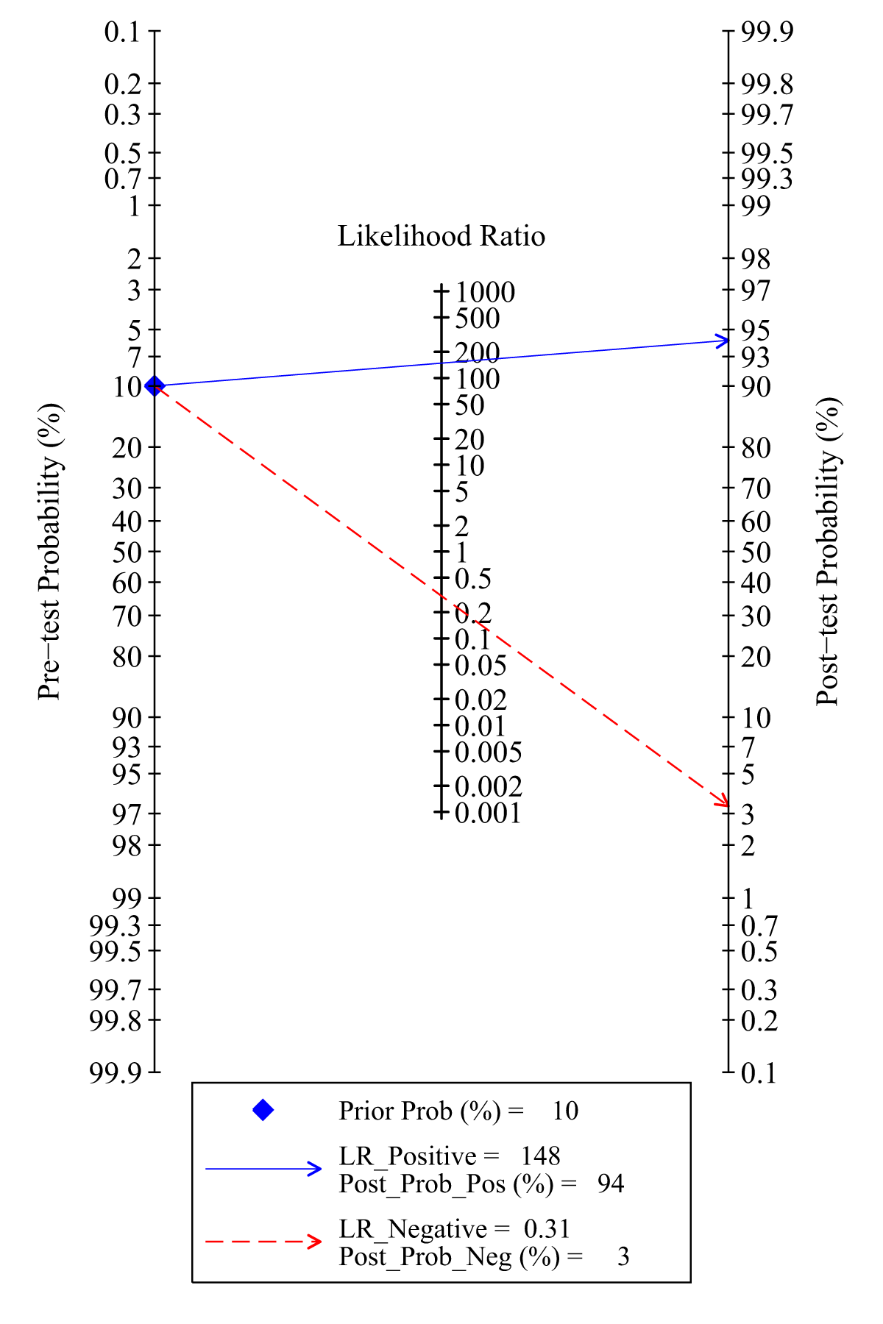

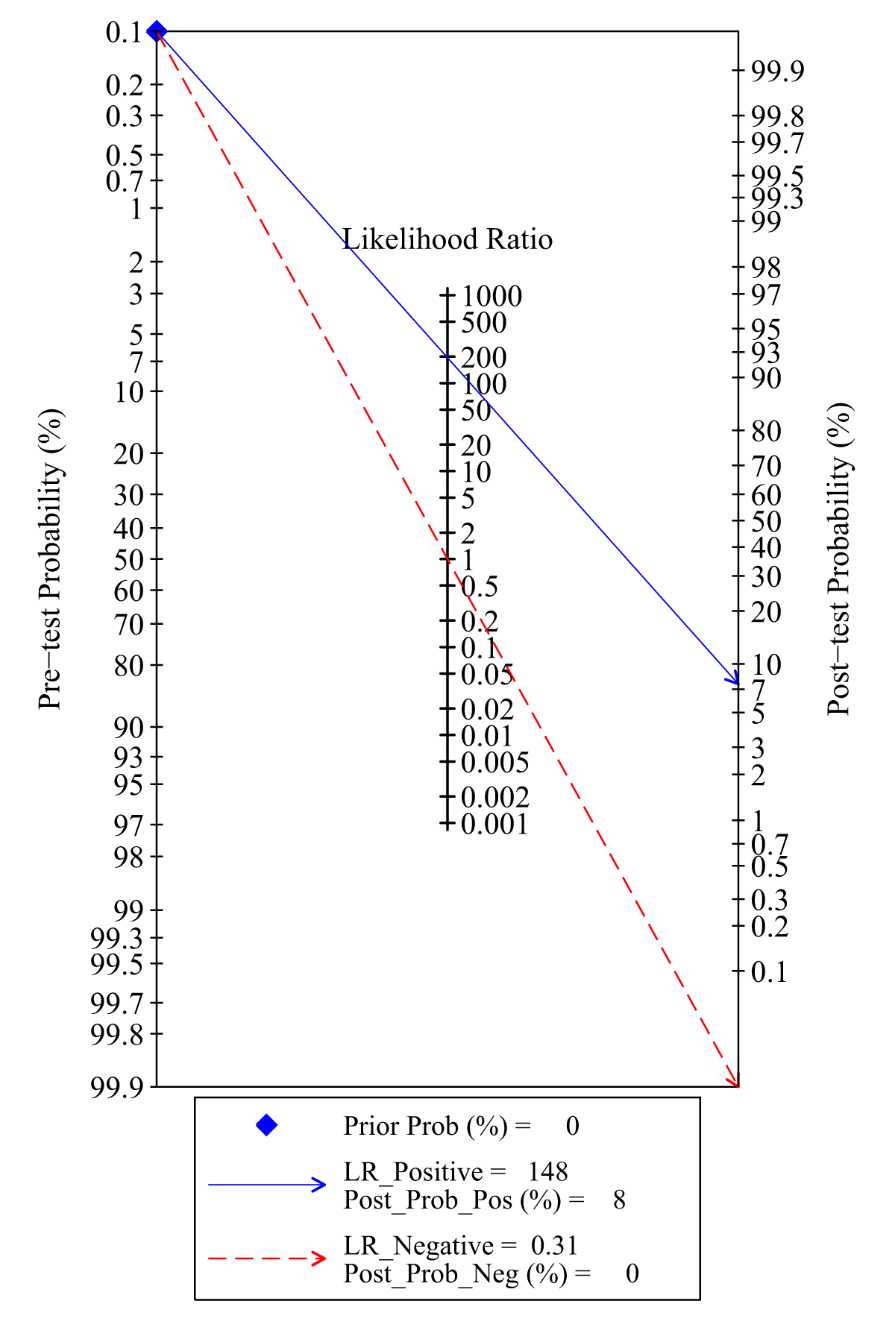
**Supplementary material 2**

A B

Figure A shows the Feigen chart of the actual incidence rate in China, and Figure B shows the Feigen chart when the prevalence rate is 10%. Feigen diagram shows that mNGS is not suitable for tuberculosis screening experiment at present, and it is applicable only in areas with high prevalence rate.

| Subgroup | sensitivity | P value | I2 | specificity | P value | I2 |
| --- | --- | --- | --- | --- | --- | --- |
| Pulmonary Specimens | 0.75[0.63 - 0.85] | <0.01 | 87.93[81.39 - 94.48] | 1.00[0.98 - 1.00] | 0.02 | 57.42 [25.85 - 88.99] |
| Specimen species  (BALF) | 0.75[0.55-0.88] | <0.01 | 91.45[86.17 - 96.72] | 0.99[0.98 - 1.00] | 0.08 | 48.84 [1.56 - 96.13] |
| Extrapulmonary Specimens | 0.61[0.47 - 0.74] | <0.01 | 87.58[81.48 - 93.67] | 0.99[0.97 - 1.00] | <0.01 | 66.02 [44.32 - 87.72] |
| Specimen species  (CSF) | 0.58[0.39-0.75] | <0.01 | 87.01[78.01 - 96.01] | 0.99[0.93 - 1.00] | 0.01 | 66.52 [37.39 - 95.65] |
| Sequencing platform(BGISEQ) | 0.64[0.51 - 0.75] | <0.01 | 87.93 [81.74 - 94.11] | 0.99[0.98 - 1.00] | 0.01 | 59.34 [31.00 - 87.68] |
| Sequencing  platform(BGISEQ-50) | 0.69[0.56 - 0.79] | <0.01 | 86.08[77.07 - 95.09] | 0.99[0.98 - 1.00] | 0.36 | 8.49 [0.00 - 100.00] |
| Sample size  (total<100) | 0.78[0.59 - 0.90] | <0.01 | 87.03[77.07 - 97.00] | 0.99[0.90 - 1.00] | 0.66 | 0.00 [0.00 - 100.00] |
| Sample size  (total>100) | 0.66[0.53 - 0.76] | <0.01 | 89.15[84.26 - 94.04] | 0.99[0.98 - 1.00] | <0.01 | 73.26 [57.86 - 88.66] |
| Diagnostic criteria(reads=1) | 0.73[0.57 - 0.84] | <0.01 | 94.00[91.62 - 96.38] | 1.00[0.98 - 1.00] | <0.01 | 82.43 [72.94 - 91.92] |
| Study type  Retrospective | 0.72[0.63 - 0.80] | <0.01 | 89.22[84.57 - 93.87] | 1.00[0.99 - 1.00] | <0.01 | 62.85 [40.61 - 85.09] |
| Study type  Prospective | 0.58[0.26 - 0.85] | <0.01 | 93.17[88.13 - 98.20] | 0.99[0.93 - 1.00] | 0.01 | 76.01[51.74 -100.00] |

**Table 1: Subgroup analysis summary**

Four or more studies were required to complete the subgroup analysis
